# The influence of mandibular third molar classification on fixation stability of mandibular angle fractures: a biomechanical finite element analysis

**DOI:** 10.1186/s12903-026-09329-9

**Published:** 2026-07-20

**Authors:** En-shi Jiang, Qing Cui, Hui Yang, Lili Xu, Yichuan Wang, Boyu Wang, Yixin Cui, Ruize Tang, Jingxu Li

**Affiliations:** 1 Department of Stomatology, Affiliated Hospital of Yanbian University(Yanbian University Hospital), Yanji, Jilin 133000 China; 2 Yanbian University Medical College, Jilin, 133000 China; 3 School of Stomatology, Baicheng Medical College, Baicheng, Jilin 137000 China

**Keywords:** Mandibular angle fracture, Finite element analysis, Mandibular third molar, Third molar retention, Champy fixation, Biomechanical stability, Splinting effect

## Abstract

**Background:**

The mandibular third molar (MTM) is frequently present in mandibular angle fractures treated with the Champy technique. Clinical research on MTM management has focused predominantly on postoperative complications, and several studies have suggested that retention may confer a stabilizing effect on the fracture — the so-called “splinting effect” proposed by Wolujewicz. However, this stabilizing role has never been quantitatively verified at the biomechanical level, and the geometric feature of the retained tooth responsible for this effect has not been identified. The aim of this theoretical biomechanical study was to test, by means of finite element analysis (FEA), whether a retained MTM improves the mechanical stability of Champy fixation across a range of impaction types, and to identify the key geometric determinant of any such effect.

**Methods:**

A mandibular finite element model was constructed from the cone-beam computed tomography data of an adult volunteer. Ten impaction configurations were established by combining three Pell and Gregory depth positions (A, B, C) with four Winter angulations (mesioangular, distoangular, vertical, horizontal). For each configuration, an MTM-retained and an MTM-extracted model were created, yielding 20 models in total. All models shared identical boundary conditions, material properties, and loading (bilateral molar clenching, 230 N). The primary outcome was interfragmentary displacement; secondary outcomes included plate and screw von Mises stress and proximal cortical bone maximum principal strain. Six candidate geometric variables of the retained tooth were measured and related to the stabilizing contribution (the percentage increase in displacement upon extraction) using descriptive correlation analysis.

**Results:**

In all ten impaction configurations, the MTM-retained model exhibited a smaller interfragmentary displacement than its extracted counterpart. Extraction increased displacement by 68%–172%, and this pattern was corroborated by consistent increases in plate stress (26%–61%), screw stress (23%–55%), and cortical bone strain (10%–47%). Among the geometric variables examined, the trans-fracture cross-sectional area of the tooth — the area of its cross-section at the fracture plane — showed the strongest association with the stabilizing contribution (*r* = 0.86, ρ = 0.79), whereas volume-based measures, including mesial segment volume (*r* = 0.14), were substantially weaker. Impaction depth and angulation did not act as independent determinants; configurations spanning all three depth positions followed a common cross-sectional-area trend.

**Conclusions:**

Under the conditions of this finite element model, retention of the MTM was associated with improved biomechanical stability of Champy fixation in every impaction configuration studied. The magnitude of this stabilizing effect was governed primarily by the cross-sectional area of the tooth at the fracture plane rather than by the conventional angulation- or depth-based classifications, which appeared to act only indirectly through the cross-section they produce. These findings are theoretical and derived exclusively from computational simulation; they require corroboration by in vitro testing and prospective clinical studies before informing clinical decision-making.

**Supplementary Information:**

The online version contains supplementary material available at https://doi.org/10.1186/s12903-026-09329-9.

## Background

 Mandibular angle fractures are among the most common mandibular fractures [1], and the Champy technique is a widely used method for their treatment. Multiple systematic reviews and meta-analyses have demonstrated that the Champy technique is superior to alternative fixation methods, particularly with regard to postoperative complication rates, and it is currently regarded as one of the most favorable treatment options [2–4]. When the Champy technique is used for fixation of a mandibular angle fracture, the mandibular third molar (MTM) is an important consideration.

Previous studies have shown that the presence of an MTM significantly increases the risk of mandibular angle fracture [5, 6]; moreover, an MTM is present in a high proportion of mandibular angle fractures, frequently lying in or near the line of fracture [7].

To date, much research on managing an MTM in the line of fracture has focused on the effect of retention versus extraction on postoperative complications—both infectious (e.g., inflammation and abscess) and mechanical (e.g., plate fracture and screw loosening); some studies have also addressed the management of angle fractures with a concomitantly injured MTM [8–10]. The mechanical stability of the fixation is a prerequisite for fracture healing [11]; however, these studies have largely approached the problem from a clinical management perspective, and the influence of an MTM in the line of fracture on the postoperative mechanical stability of the fixation has rarely been investigated.

In a retrospective radiographic analysis of mandibular angle fractures, Wolujewicz [12] observed that mesioangular or horizontally impacted MTMs were associated with smaller fragment displacement and required only simple treatment, whereas vertically impacted MTMs showed greater displacement. On this basis, he proposed the “splinting effect” theory—that an MTM in the line of fracture may act like a splint, stabilizing the fracture fragments. However, this theory was derived from a small-sample retrospective clinical observation (*n* = 47), has not been quantitatively investigated at the biomechanical level, and its underlying mechanism remains unclear.

Using finite element analysis (FEA), the present study compared the fixation stability of mandibular angle fractures between MTM-retained and MTM-extracted conditions within each impaction configuration, in order to test the splinting effect proposed by Wolujewicz — namely, whether an MTM retained in the line of fracture exerts a stabilizing effect on the fixation — and to investigate its underlying biomechanical mechanism. Building on this, and following the Winter classification (based on angulation) used by Wolujewicz, we hypothesized that MTMs with different Winter classifications exert different effects on fixation stability. We further introduced the Pell and Gregory classification (based on impaction depth) and proposed an additional hypothesis that the impaction depth of the MTM likewise influences fixation stability.

## Methods

An adult female volunteer was selected based on the following inclusion criteria: no malocclusion, no history of orthodontic treatment, no metallic restorations in the mandibular molar region, and presence of a mandibular third molar. Cone-beam computed tomography (CBCT) scanning was performed on the volunteer’s maxillofacial region, and the imaging data were exported in Digital Imaging and Communications in Medicine (DICOM) format. This study was approved by the Ethics Committee of the affiliated hospital (Approval No. 2024210), and the volunteer signed written informed consent. This study adhered to the World Medical Association Declaration of Helsinki governing medical research.

### Model establishment

Mandibular Reconstruction: The DICOM data were imported into Mimics 26.0 software (Materialise, Belgium). Three-dimensional reconstruction was completed using the “New Mask” function, followed by the “Split Mask” command to separate the dental crowns from the mandibular model. The models were exported in standard tessellation language (STL) format using the “Calculate Part” command.

Mandibular Dentition Reconstruction: BlueSkyBio Plan 4.1.1 software (Blue Sky Bio, USA) was used for automatic tooth segmentation via the “Automatic Tooth Segmentation” function, and the complete dentition model was exported in STL format. This method demonstrated superior anatomical fidelity and workflow efficiency compared to manual segmentation in Mimics.

**Fig. 1:**
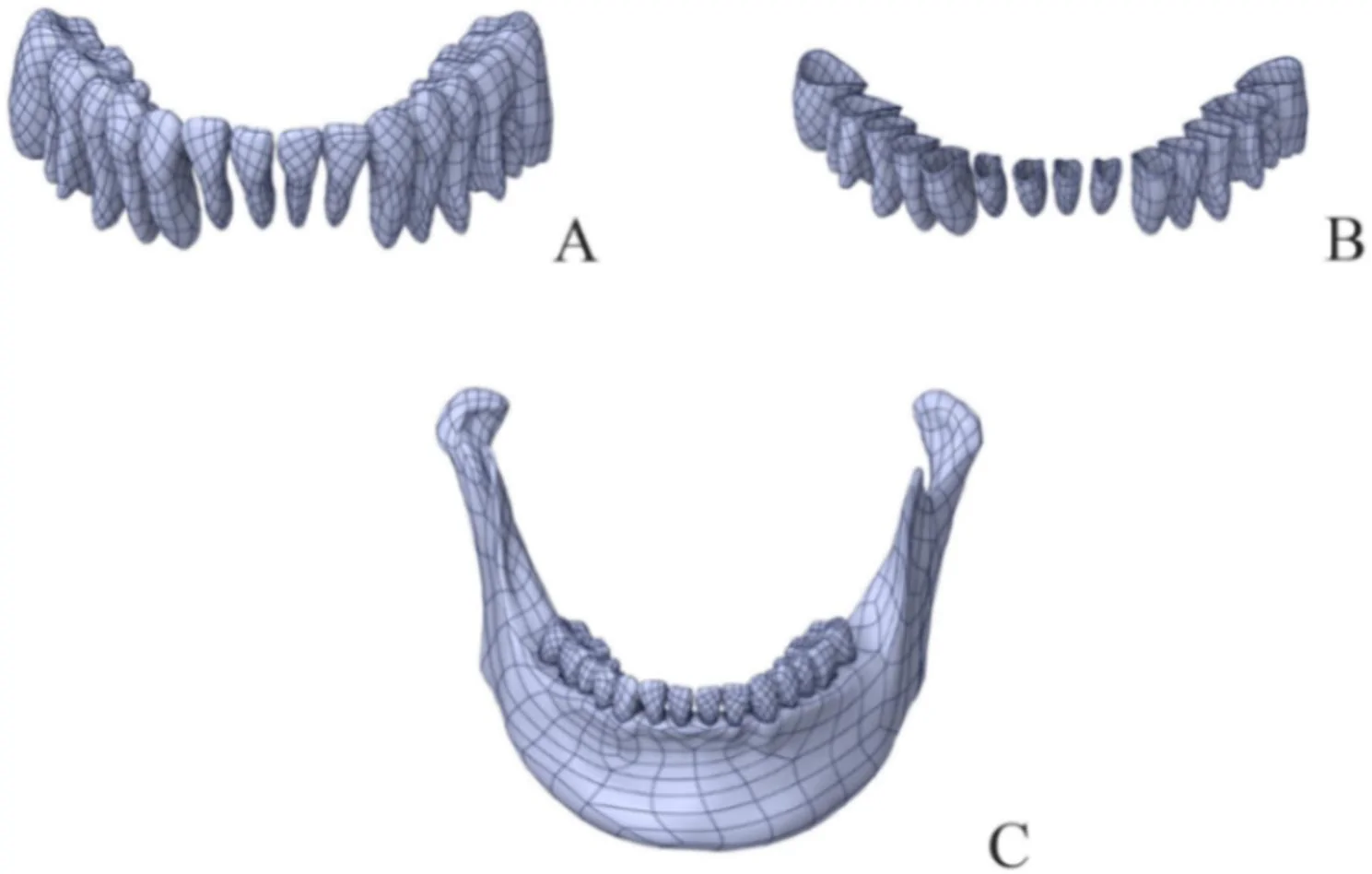
Complete three-dimensional mandibular model. **A** Dentition model. **B** Periodontal ligament model. **C** Complete mandibular model with dentition

Model Registration: The mandibular model, dental crown model, and complete mandibular dentition model were imported into 3-Matic 18.0 (Materialise, Belgium). Using the “N Points Registration” method, with cusp tips and axial surface high points of the dental crowns as registration landmarks, the dentition was precisely aligned to its correct anatomical position on the mandible (Fig. 1).

### Finite element model construction

Periodontal Ligament and Cancellous Bone Modeling: The periodontal ligament thickness was set at 0.25 mm. The dentition model was imported into Geomagic Warp 2021 (Raindrop, USA), and the tooth surfaces were uniformly offset outward by 0.25 mm using the “Offset” function. In the SpaceClaim module of Ansys 2021 R1 (ANSYS, USA), the alveolar socket, periodontal ligament, and cancellous bone solid models were constructed through Boolean operations.

#### Fracture line establishment 

**Fig. 2:**
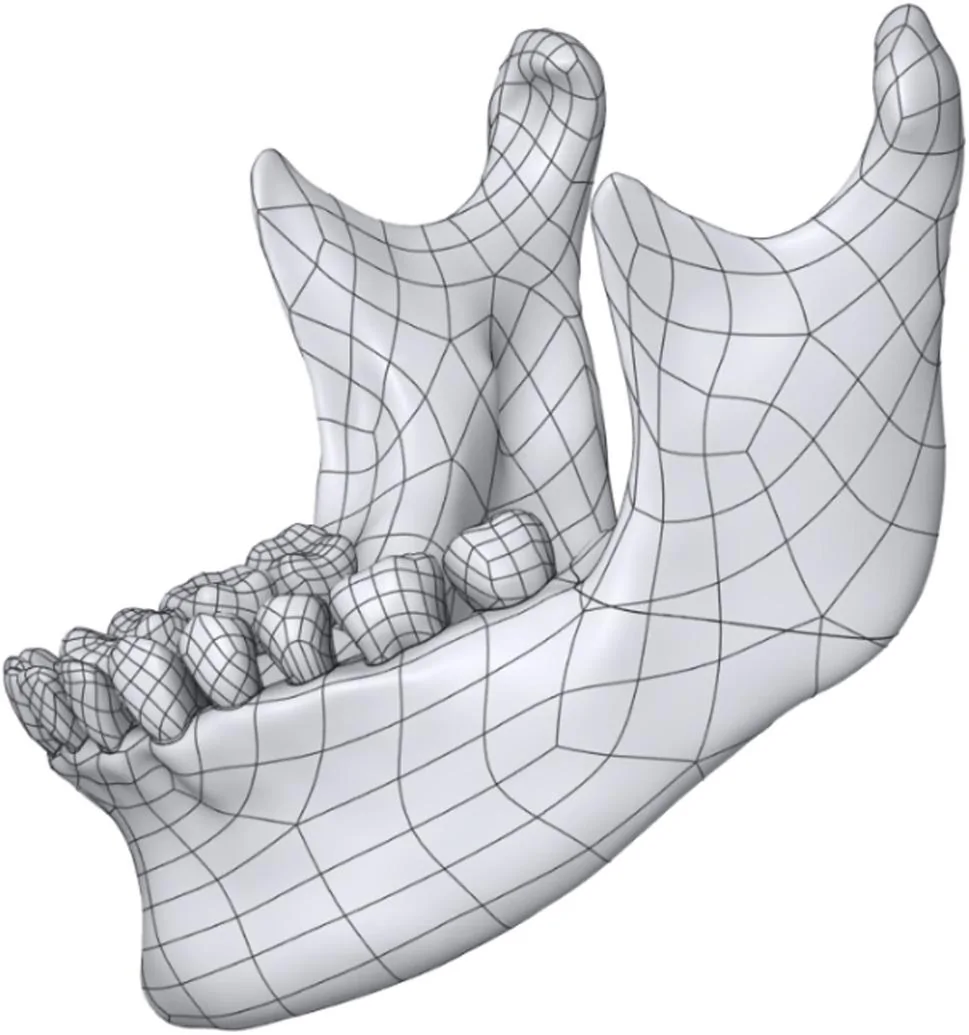
Fracture line establishment on the mandibular angle

Within the “Design” module of SpaceClaim, a reference plane was established along the direction of the mandibular angle fracture line, and the “Combine” function was used to complete fracture line modeling (Fig. 2).

Miniplate modeling

**Fig. 3:**
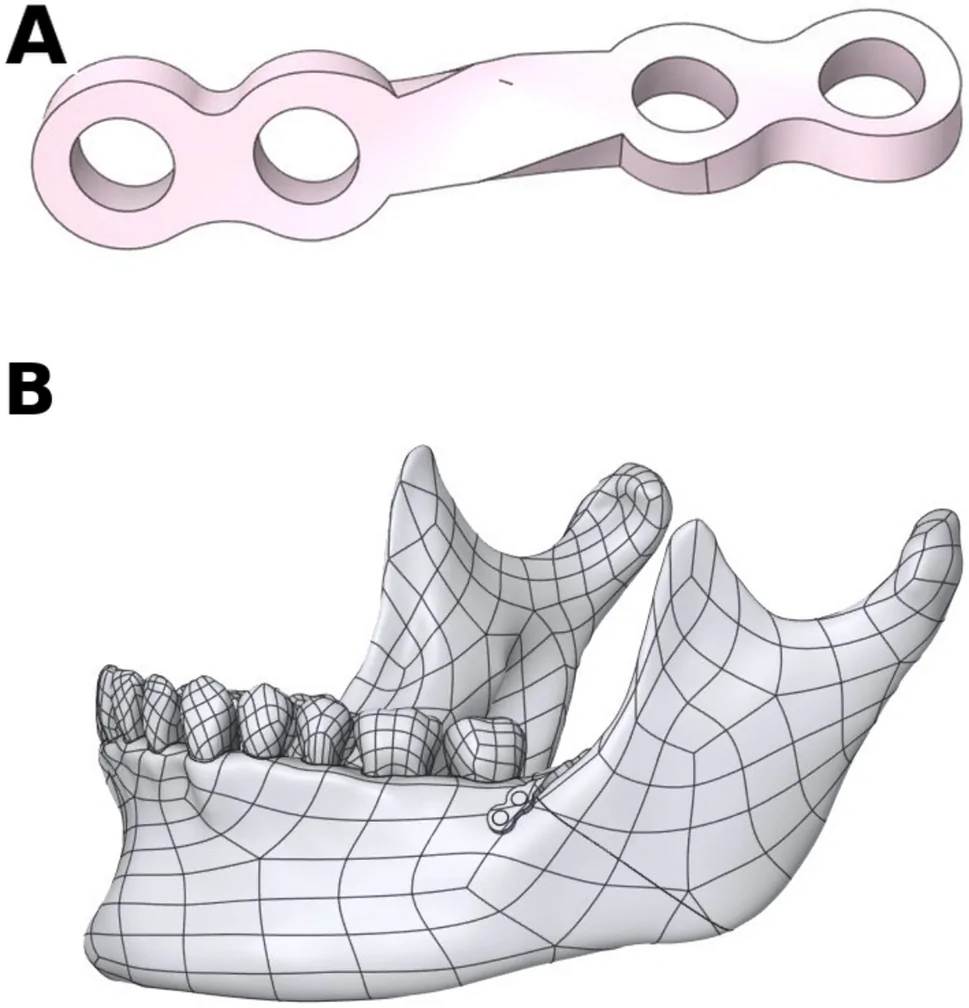
Three-dimensional models of the miniplate fixation system. **A** Four-hole miniplate and screw models. **B** Miniplate fitted to the bone surface along the external oblique ridge

Three-dimensional modeling was performed based on the standard parameters of a DePuy Synthes (Johnson & Johnson, USA) four-hole miniplate (thickness: 1 mm, screw hole diameter: 2 mm). A reference plane was established along the fixation direction, a two-dimensional profile was drawn and projected onto the bone surface via surface projection to ensure conformity with the bone surface geometry. The surface was duplicated with a 1 mm offset to construct the thickness feature, generating the solid miniplate model. Boolean operations were used to create screw channels and establish the contact relationships between screws and bone. The miniplate was positioned along the external oblique ridge in accordance with the Champy technique (Fig. 3).

MTM model construction

**Fig. 4:**
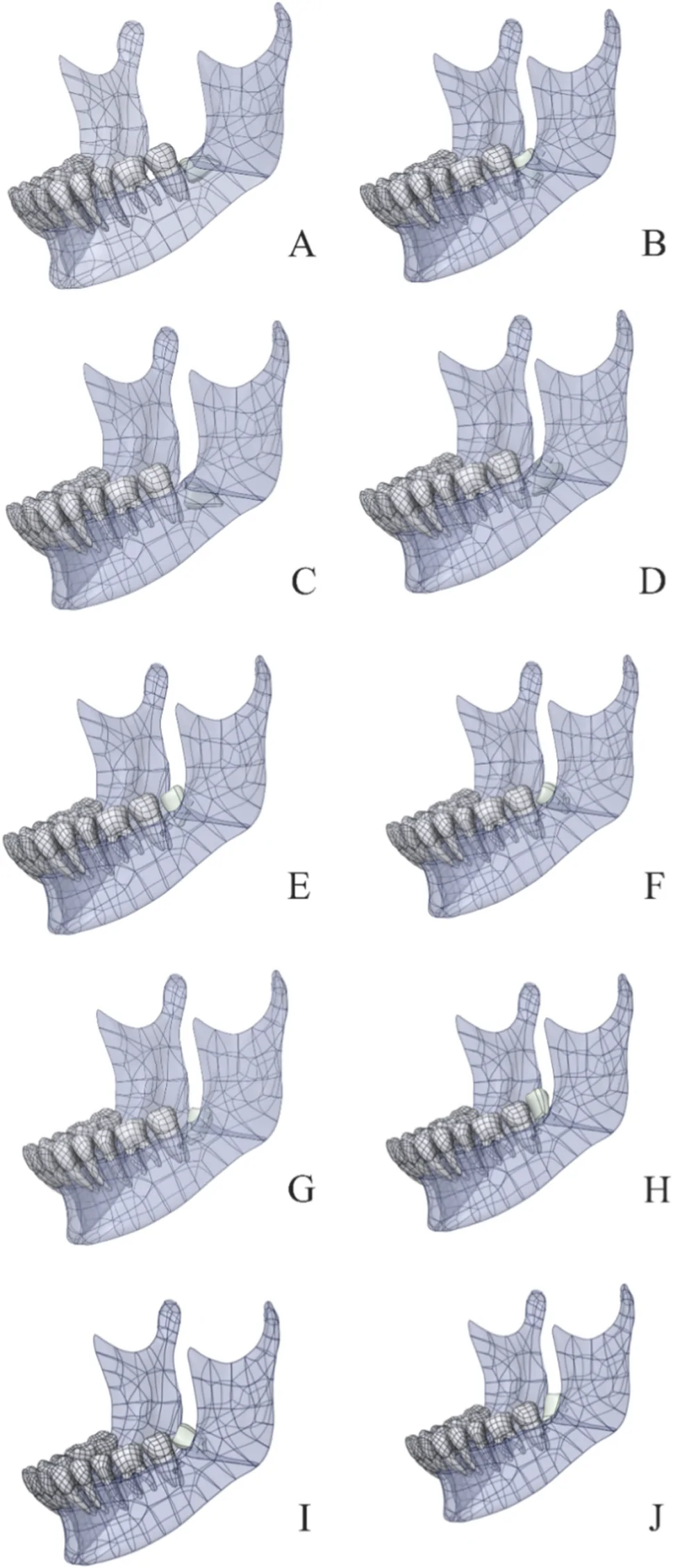
MTM-retained experimental groups according to the Pell and Gregory/Winter classification. **A** C-H (Position C, horizontal). **B** C-V (Position C, vertical). **C** C-M (Position C, mesioangular). **D** C-D (Position C, distoangular). **E** B-V (Position B, vertical). **F** B-M (Position B, mesioangular). **G** B-D (Position B, distoangular). **H** A-V (Position A, vertical). **I** A-M (Position A, mesioangular). **J** A-D (Position A, distoangular)

**Fig. 5:**
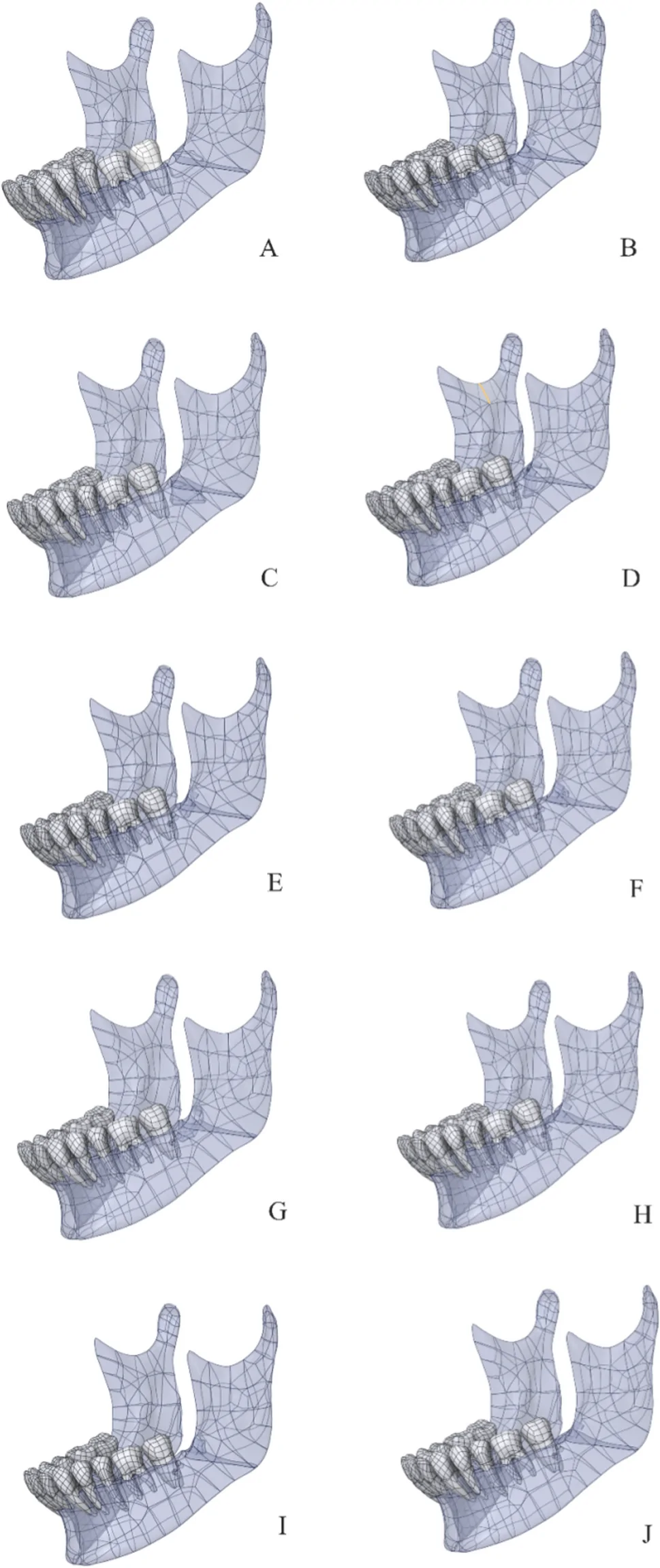
MTM-extracted experimental groups according to the Pell and Gregory/Winter classification. **A** C-H (Position C, horizontal). **B** C-V (Position C, vertical). **C** C-M (Position C, mesioangular). **D** C-D (Position C, distoangular). **E** B-V (Position B, vertical). **F** B-M (Position B, mesioangular). G B-D (Position B, distoangular). **H** A-V (Position A, vertical). **I** A-M (Position A, mesioangular). **J** A-D (Position A, distoangular)

According to the Pell and Gregory classification, which categorizes impaction depth by the position of the highest point of the MTM relative to the occlusal plane and cervical line of the second molar (Position A, at or above the occlusal plane; Position B, below the occlusal plane but above the cervical line; Position C, below the cervical line), and the Winter classification, which categorizes the angulation of the MTM long axis relative to the second molar long axis as mesioangular (M), distoangular (D), vertical (V), or horizontal (H), ten impaction configurations of the MTM were established. Each configuration is abbreviated by combining its Pell and Gregory position (A, B, or C) with its Winter angulation (M, D, V, or H); for example, C-H denotes a horizontally impacted MTM in Position C. For each configuration, an MTM-retained model and an MTM-extracted model were created, yielding a total of 20 models (Figs. 4 and 5).

### Finite element analysis

#### Material properties

All tissues and materials were defined as homogeneous, isotropic, and linearly elastic materials. Material parameters were set according to previously published studies [13, 14] (Table 1).

**Table 1:** Material properties

Material	Elastic Modulus (MPa)	Poisson’s Ratio
Teeth	18,600	0.31
Periodontal ligament	68	0.45
Cortical bone	13,700	0.3
Cancellous bone	1370	0.3
Miniplate and screws	110,300	0.31

### Contact and boundary conditions

**Fig. 6:**
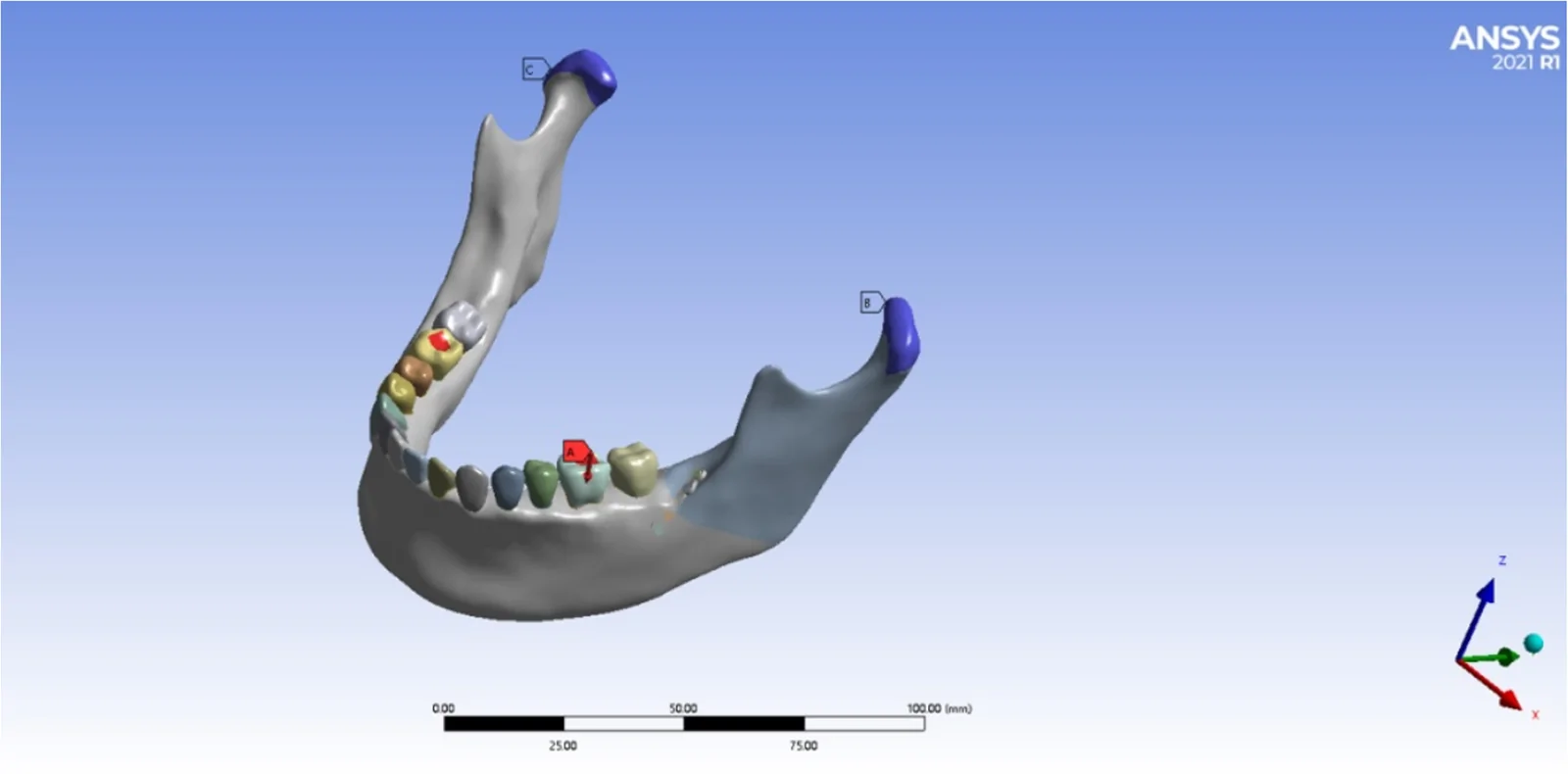
Boundary conditions and loading setup. Both condyles were fully constrained in all degrees of freedom (fixed support), representing the static intercuspal clenching condition. A vertical downward occlusal force of 230 N was applied bilaterally to the first molars

The bone surfaces on both sides of the fracture line were set as frictional contact with a friction coefficient of 0.3. The screw–bone interface was modeled as bonded contact. Both condyles were fully constrained in all degrees of freedom (fixed support), representing the static intercuspal clenching (centric occlusion) condition, in which the condyles are seated within the glenoid fossae and remain essentially stationary. A vertical downward occlusal force of 230 N [15] was applied to the bilateral first molars, with the midpoint of the mandible taken as the origin of the coordinate system (Fig. 6).

### Mesh generation and convergence analysis

Mesh generation was performed on all components of the model. A mesh convergence analysis was conducted by progressively refining the mesh density to ensure that the computational results were not significantly affected by mesh size. Convergence was considered achieved when the variation in the key metric (relative displacement of fracture fragments) between two successive mesh refinements was less than 5%, and the corresponding mesh density was selected for the final computation.

#### Mesh generation and convergence analysis

**Fig. 7:**
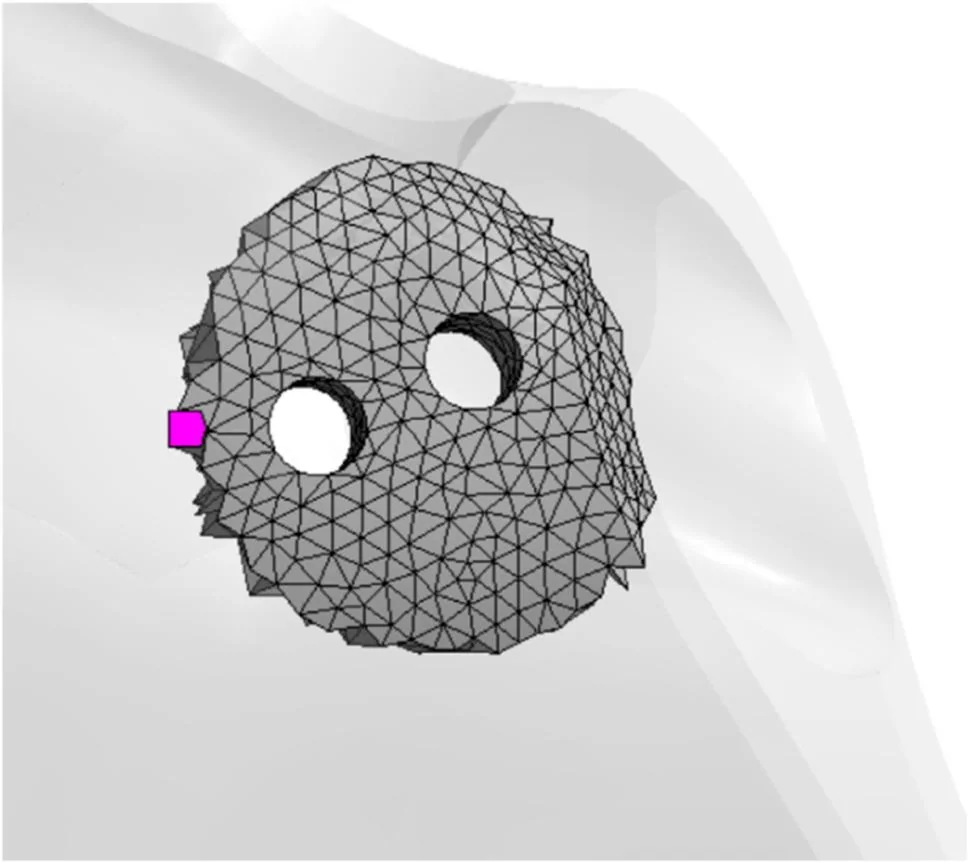
Locally refined finite element mesh of the distal (tooth-bearing) mandibular segment. The finite element mesh of the distal (tooth-bearing) segment of the mandible, shown with the adopted local element size of 0.6 mm in the region surrounding the line of fracture. A global bone element size of 2.5 mm was used, with local refinement to 0.6 mm around the fracture to resolve the stress and strain distribution in the region of interest

**Fig. 8:**
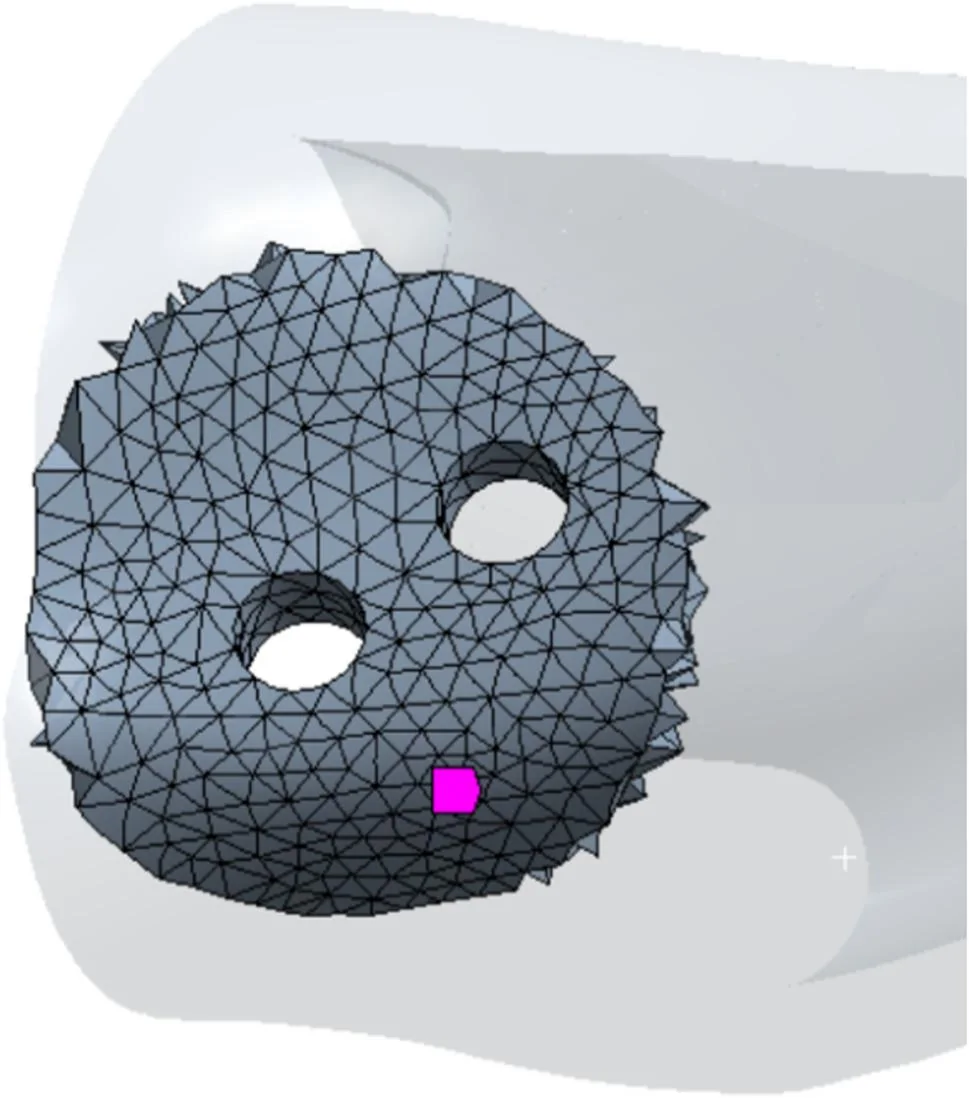
Locally refined finite element mesh of the proximal (ramus–condyle) mandibular segment. The finite element mesh of the proximal (ramus–condyle) segment of the mandible, shown with the same adopted local element size of 0.6 mm in the region surrounding the line of fracture. The global and locally refined element sizes correspond to those used in Fig. 7

**Fig. 9:**
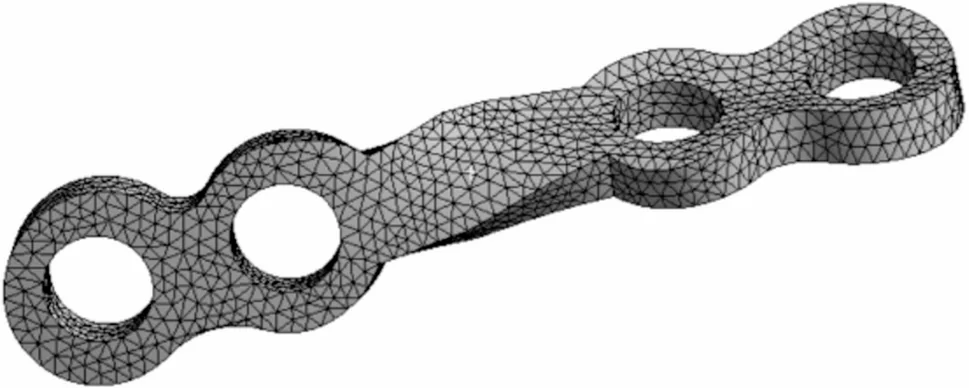
Finite element mesh of the titanium miniplate. The finite element mesh of the four-hole titanium miniplate, generated with an element size of 0.2 mm. This size was selected on the basis of the hardware mesh convergence analysis, in which the plate von Mises stress (element-averaged) varied by no more than 1.5% across the element sizes evaluated

**Fig. 10:**
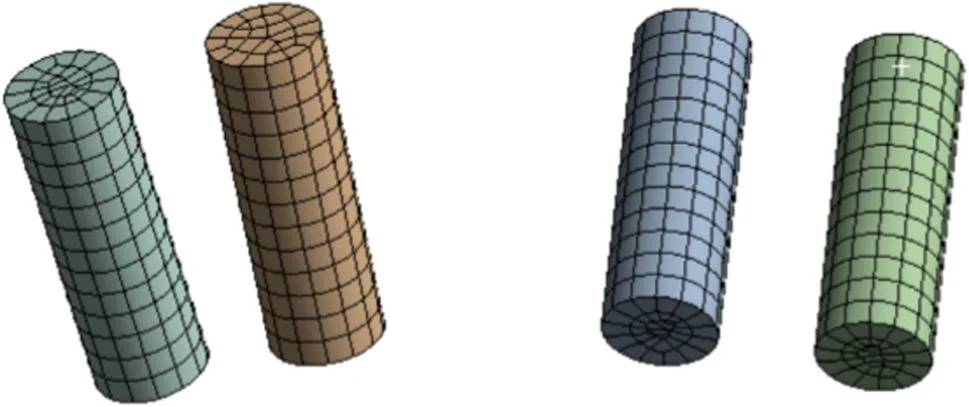
Finite element mesh of the fixation screws. The finite element mesh of the fixation screws, generated with an element size of 0.4 mm. This size was selected on the basis of the hardware mesh convergence analysis, in which the screw von Mises stress (element-averaged) varied by no more than 3.8% across the element sizes evaluated

All model components were discretized with ten-node tetrahedral elements (SOLID187). A mesh convergence study was performed on a representative model (C-H configuration, MTM-retained) to verify that the computational results were independent of mesh density; convergence was defined as less than 5% variation in the monitored quantity between successive refinements. For the bone, a global element size of 2.5 mm was assigned, and a local refinement zone was applied around the fracture region; the local element size was progressively refined through 0.9, 0.6, and 0.4 mm while the hardware mesh was held at its final sizes. Cortical bone maximum principal strain (element-averaged) varied by less than 1.2% across all refinements (Table 2), and a local refinement size of 0.6 mm was adopted (Figs. 7 and 8). For the titanium plate and screws, four plate–screw element-size combinations were evaluated while the bone mesh was held at its final sizes; plate and screw von Mises stress (element-averaged) varied by no more than 1.5% and 3.8%, respectively (Table 3), and element sizes of 0.2 mm (plate, Fig. 9) and 0.4 mm (screws, Fig. 10) were selected. Because stress and strain are inherently more sensitive to mesh density than displacement, the convergence of these quantities ensures that the primary outcome measure—interfragmentary displacement—was likewise mesh-independent. All stress and strain results in this study were reported as element-averaged values; peak nodal values at geometric discontinuities and contact edges, where stress singularities arise under linear-elastic assumptions, were excluded. The final mesh parameters for the representative model are summarized in Table 4.

**Table 2:** Mesh convergence: bone local refinement (C-H configuration)

Refinement zone size (mm)	Max. principal strain (µε)	Relative change (%)
0.9	372	—
0.6^a^	368	−1.1
0.4	372	+ 0.9

Convergence assessed on a representative model (C-H, MTM-retained). Global bone element size fixed at 2.5 mm; hardware mesh at final sizes (plate 0.2 mm, screw 0.4 mm). Monitored quantity: cortical bone maximum principal strain (element-averaged)

ᵃSelected for the production mesh. All sequential changes < 5%, confirming mesh convergence; 0.6 mm was adopted as the converged local refinement size

**Table 3:** Mesh convergence: hardware (C-H configuration)

Plate size (mm)	Screw size (mm)	Plate von Mises stress (MPa)	Screw von Mises stress (MPa)
0.3	0.5	35.82	10.34
0.2^a^	0.4^a^	35.27	9.96
0.15	0.3	35.34	9.97
0.1	0.2	35.56	10.13

Convergence assessed on a representative model (C-H, MTM-retained). Bone mesh at final sizes (global 2.5 mm, local refinement 0.6 mm). Monitored quantities: plate and screw von Mises stress (element-averaged)

ᵃSelected for the production mesh. Maximum variation across all four configurations: plate ≤ 1.5%, screw ≤ 3.8%

**Table 4:** Final mesh parameters (C-H configuration)

Component	Element type	Element size (mm)	Nodes	Elements
Cortical bone	SOLID187 (Tet10)	2.5 (local 0.6)	66,657	35,915
Cancellous bone	SOLID187 (Tet10)	2.5 (local 0.6)	58,909	34,570
Titanium plate	SOLID187 (Tet10)	0.2	64,268	41,615
Screws (×4)	SOLID187 (Tet10)	0.4	10,794	2,176
**Total**	—	—	**200**,**628**	**114**,**276**

Values for a representative model (C-H, MTM-retained). Cortical bone values: sum of anterior and posterior cortical bodies. Cancellous bone values: sum of anterior and posterior cancellous bodies. Screw values: sum of four individual screws. Tooth elements are not included; total node and element counts varied slightly across the 20 models owing to differences in MTM geometry

#### Outcome measures

The primary outcome was interfragmentary displacement, defined as the resultant relative displacement between the proximal (ramus-condyle) and distal (tooth-bearing) fracture segments under load. For each model, directional deformation components (X, Y, and Z) were extracted as face-averaged values over the entire fracture cross-section (cortical and cancellous bone combined) on each segment; the resultant displacement was then computed as δ = √[(ΔUx)² + (ΔUy)² + (ΔUz)²], where ΔU represents the component-wise difference between the distal and proximal fracture surfaces. The same measurement surfaces and extraction protocol were applied uniformly to all 20 models.

The stabilizing contribution of the retained MTM in each impaction configuration was quantified as the displacement increase upon extraction: (δ_extracted − δ_retained) / δ_retained × 100%. A larger displacement increase indicates a greater stabilizing contribution of the MTM in that configuration.

Secondary outcomes included titanium plate von Mises stress, screw von Mises stress, and cortical bone maximum principal strain at the proximal segment. All stress and strain values were reported as element-averaged quantities; peak nodal values at geometric discontinuities were excluded. These measures served as independent cross-validation of the primary outcome from the perspectives of hardware load sharing and peri-fracture bone deformation, respectively.

All outcomes were evaluated through relative comparisons (MTM-retained versus MTM-extracted within each configuration) and trend analyses across configurations, rather than against any absolute displacement threshold. Given the deterministic nature of finite element analysis and the systematically designed — rather than randomly sampled — set of ten impaction configurations, correlation coefficients (Pearson r and Spearman ρ) are reported as descriptive measures of association strength, not as inferential hypothesis tests; no p-values are claimed.

### Geometric variables of the retained third molar

To examine which geometric features of the retained MTM, if any, were associated with its stabilizing contribution (quantified as the displacement increase upon extraction), six candidate geometric variables were defined for each impaction configuration and measured directly from the model geometry in SpaceClaim (Ansys 2021 R1). Because an identical tooth geometry was used in all ten configurations, the total tooth volume was constant across models; the variables defined below therefore characterize how this single tooth was partitioned by the fracture plane and by the surrounding bone in each configuration, rather than any difference in tooth size.

**Fig. 11:**
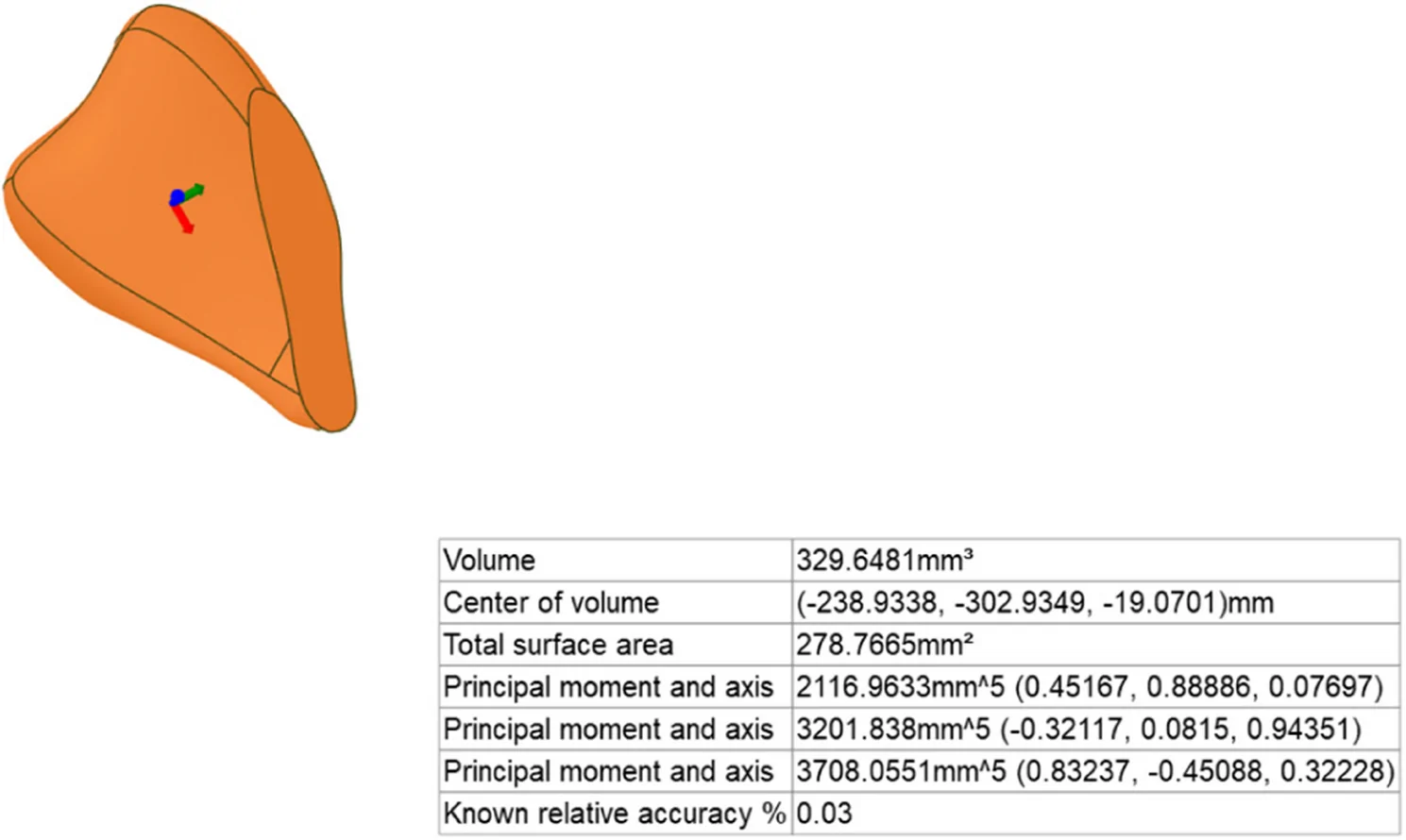
Measurement of a tooth-segment volume using the Mass Properties tool

**Fig. 12:**
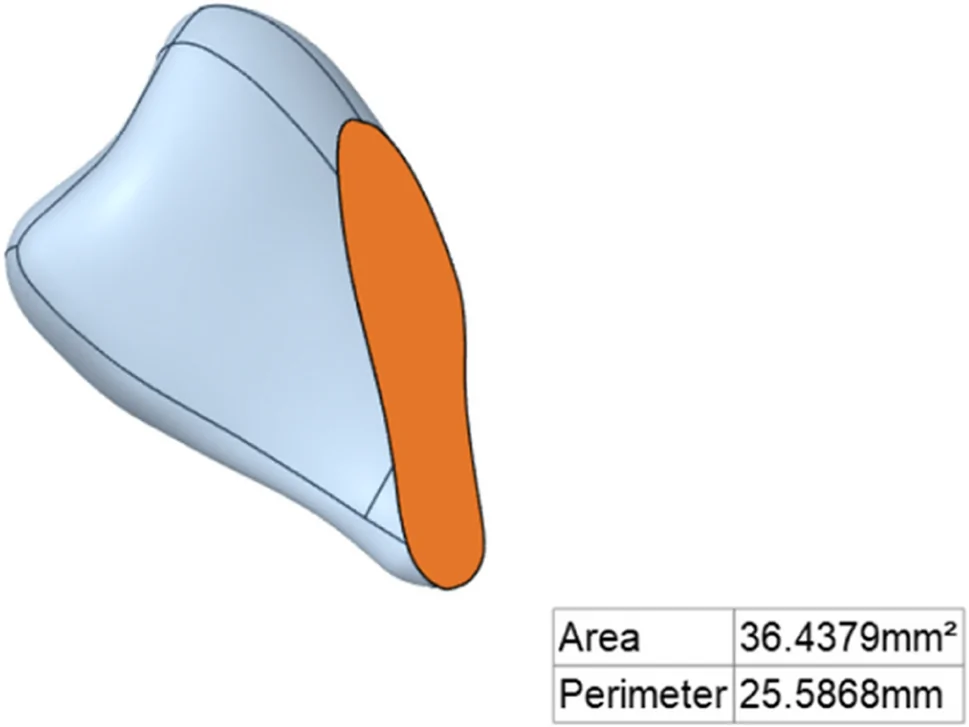
Measurement of the trans-fracture cross-sectional area using the Measure tool

For each model, the retained tooth was first separated into its intraosseous and extraosseous portions by a Boolean operation with the surrounding mandibular bone (Combine). The intraosseous portion was then divided into a mesial and a distal segment by the plane coinciding with the line of fracture (hereafter, the fracture plane), using Split Body. Because all configurations shared the same fracture geometry, an identical fracture plane was used in every model. The mesial segment of the tooth is the part lying mesial to the fracture (toward the second molar), and the distal segment of the tooth is the part lying distal to the fracture (toward the ramus). The volume of each resulting body (Fig. 11) was obtained from its mass properties (Mass Properties), and the trans-fracture cross-sectional area (Fig. 12) was measured as the area of the tooth’s cross-section on the fracture plane, read directly from the corresponding planar face (Measure).

The six geometric variables were defined as follows:

- Cross-sectional area — the trans-fracture cross-sectional area of the tooth(the area of the tooth’s cross-section on the fracture plane).
- Mesial segment volume — the volume of the mesial segment of the tooth (the intraosseous portion mesial to the fracture).
- Intraosseous volume — the total volume of the tooth lying within bone, equal to the sum of the mesial and distal tooth-segment volumes.
- Lesser-segment volume — the smaller of the two intraosseous tooth-segment volumes.
- Segment symmetry ratio — the ratio of the lesser to the greater intraosseous tooth-segment volume, ranging from 0 (markedly asymmetric) to 1 (symmetric).
- Extraosseous volume — the volume of the tooth lying outside bone.
- Volumes are reported in mm³ and the cross-sectional area in mm².

The volume of each body produced by the Boolean (Combine) and sectioning (Split Body) operations—the mesial and distal intraosseous segments and the extraosseous portion of the tooth—was obtained from its mass properties in SpaceClaim. The figure illustrates this measurement for one representative segment.

The trans-fracture cross-sectional area was measured as the area of the tooth’s cross-section on the fracture plane, read directly from the corresponding planar face with the Measure tool in SpaceClaim.

## Results

### Effect of MTM retention on interfragmentary displacement

**Fig. 13:**
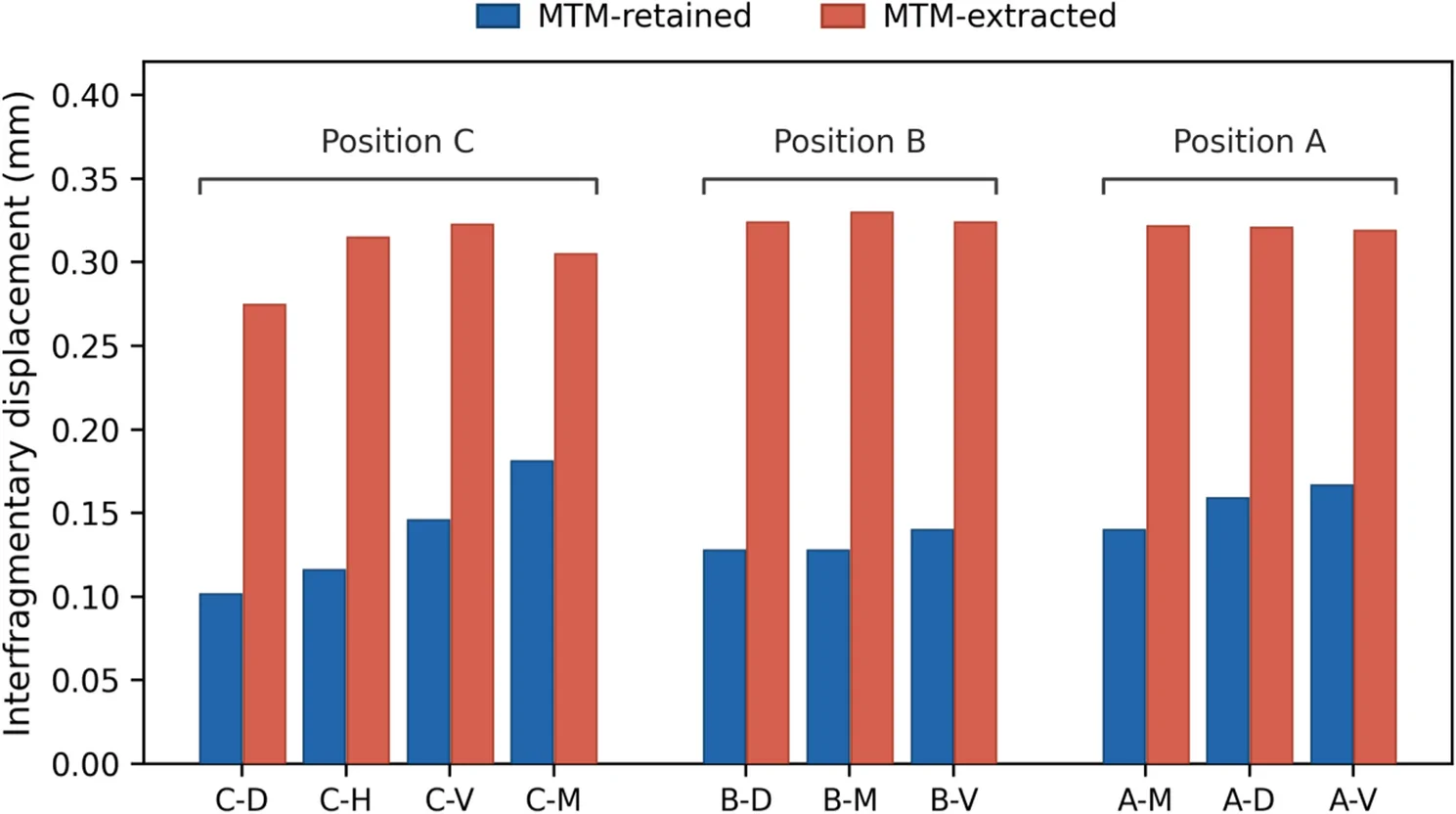
Interfragmentary displacement for each impaction configuration with and without the retained MTM. Paired bars show the interfragmentary displacement for the MTM-retained (blue) and MTM-extracted (red) models across all ten configurations, grouped by Pell and Gregory depth position. Extracted displacement was consistently higher than retained displacement in every configuration, confirming the universal stabilizing effect of the retained MTM. Among the retained models, Position A (shallow) exhibited the highest displacement values, indicating the least stable fixation despite tooth retention. Within each depth group, configurations are sorted by ascending retained displacement. Brackets above the bars denote the three depth groups

Across all ten impaction configurations, the model retaining the MTM consistently produced a smaller interfragmentary displacement than its extracted counterpart (Table 5; Fig. 13). In the retained models, displacement ranged from 0.102 mm (C-D) to 0.181 mm (C-M), whereas in the extracted models it ranged from 0.275 mm (C-D) to 0.330 mm (B-M). Extraction of the MTM increased interfragmentary displacement in every configuration without exception, by 68% (C-M) to 172% (C-H), indicating that the retained tooth conferred a stabilizing effect on the Champy fixation regardless of impaction type.

**Table 5:** Interfragmentary displacement for each impaction configuration

Configuration	Retained (mm)	Extracted (mm)	Displacement increase (%)
Position C			
C-D	0.102	0.275	171
C-H	0.116	0.315	172
C-V	0.146	0.323	122
C-M	0.181	0.305	68
Position B			
B-M	0.128	0.330	158
B-D	0.128	0.324	153
B-V	0.140	0.324	131
Position A			
A-M	0.140	0.322	130
A-D	0.159	0.321	102
A-V	0.167	0.319	91

Each configuration is designated by combining the Pell and Gregory depth position (A, B, or C) with the Winter angulation (M = mesioangular, D = distoangular, V = vertical, H = horizontal). Retained = model with the mandibular third molar (MTM) in situ; Extracted = model with the MTM suppressed. Displacement increase represents the percentage rise in interfragmentary displacement upon removal of the MTM, quantifying its stabilizing contribution. Values within each depth group are sorted by ascending retained displacement. All models share identical geometry, fracture line, fixation hardware, loading (230 N bilateral molar force), and boundary conditions (fixed support at condyles)

The baseline stability of the retained construct varied with impaction depth. Among the retained models the lowest displacements—and thus the most stable fixation—occurred in the deep (Position C) configurations C-D and C-H, while the shallow (Position A) configurations clustered at the higher end of the range, identifying Position A as the least stable group despite tooth retention. The relationship between depth and stability was not strictly monotonic: C-M, although a deep (Position C) impaction, showed the highest retained displacement of any configuration, an observation accounted for by the cross-sectional analysis below.

### Hardware stress and cortical bone strain

The same pattern was reflected in the secondary mechanical outcomes (Table 6). Relative to the retained model, extraction of the MTM increased the element-averaged plate von Mises stress in every configuration (by 26%–61%), the element-averaged screw von Mises stress in every configuration (by 23%–55%), and the element-averaged maximum principal strain in the proximal (ramus–condyle) cortical bone in every configuration (by 10%–47%). Because all three metrics changed in the same direction as interfragmentary displacement across all ten configurations, the stabilizing effect of the retained MTM was corroborated by three independent measures—construct displacement, hardware loading, and peri-implant bone strain—rather than resting on a single outcome.

**Table 6:** Percentage change in hardware stress and cortical bone strain after MTM extraction

Configuration	Plate von Mises (%)	Screw von Mises (%)	Max principal strain (%)
Position C			
C-D	+ 49	+ 28	+ 47
C-H	+ 26	+ 23	+ 29
C-V	+ 61	+ 55	+ 23
C-M	+ 48	+ 45	+ 23
Position B			
B-M	+ 47	+ 44	+ 32
B-D	+ 48	+ 46	+ 37
B-V	+ 46	+ 44	+ 29
Position A			
A-M	+ 40	+ 41	+ 28
A-D	+ 37	+ 40	+ 24
A-V	+ 29	+ 25	+ 10

Values represent the percentage increase in each metric from the MTM-retained to the MTM-extracted model. Plate and screw von Mises stress are element-averaged values over the respective hardware components. Max principal strain is the element-average maximum principal elastic strain at the cortical bone region of interest on the proximal (ramus-condyle) side of the fracture. All values are positive, indicating that MTM extraction uniformly increased hardware stress and bone strain across all configurations

### Geometric determinants of the stabilizing contribution

**Fig. 14:**
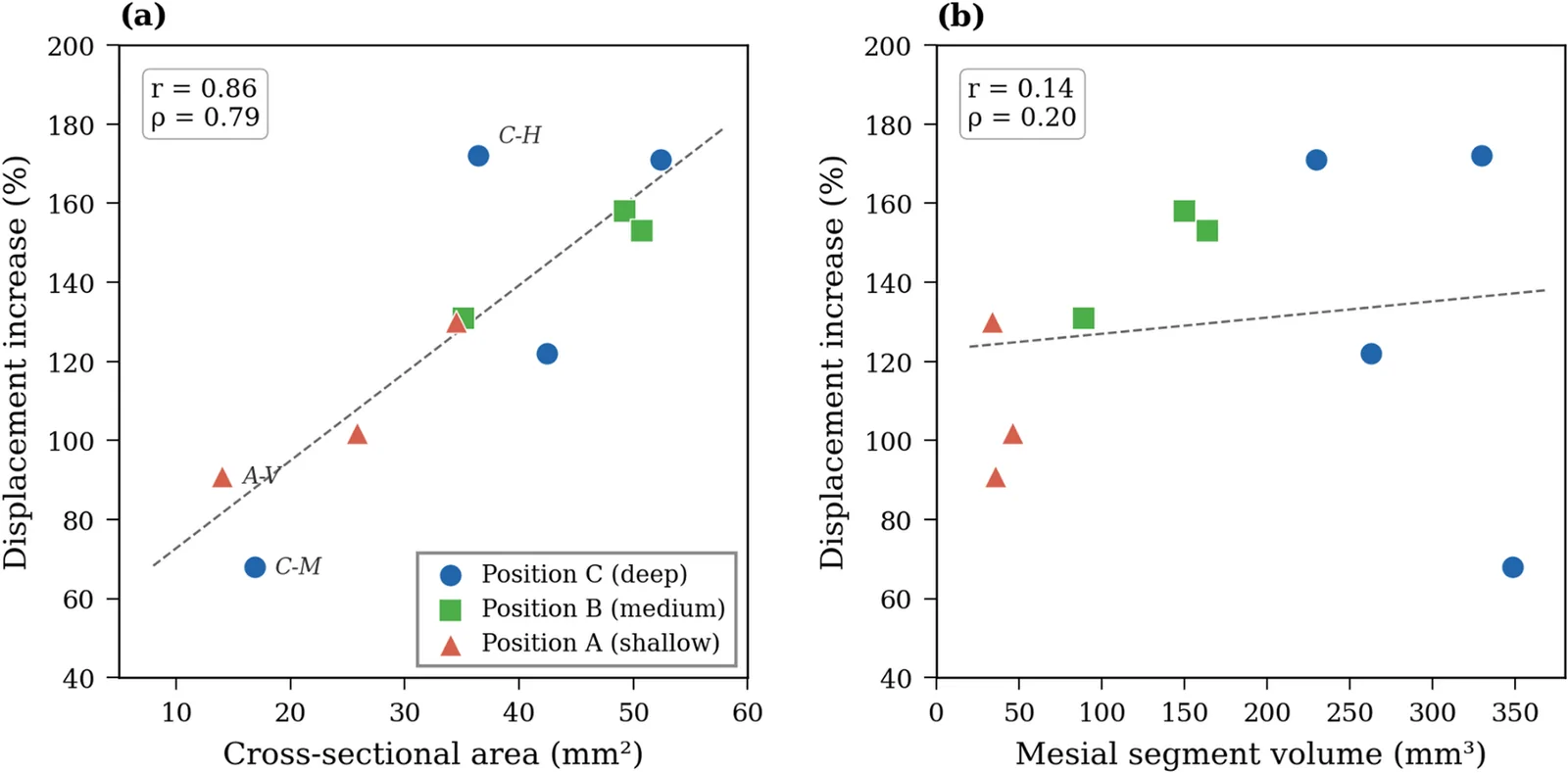
Relationship between geometric variables and the stabilizing contribution of the retained MTM. **a** Trans-fracture cross-sectional area versus displacement increase. A strong positive linear trend was observed (*r* = 0.86, ρ = 0.79), indicating that configurations with a larger cross-sectional area at the fracture plane exhibited a greater stabilizing contribution. The dashed line represents the linear regression fit. C-H (horizontal impaction) is the only notable outlier above the trend line; C-M (Position C, mesioangular) and A-V (Position A, vertical) represent the two lowest contributions despite originating from opposite ends of the depth spectrum, consistent with their similarly small cross-sectional areas. **b** Mesial segment volume versus displacement increase. No meaningful trend was observed (*r* = 0.14, ρ = 0.20), demonstrating that the overall volume of the tooth on the mesial side of the fracture did not predict its stabilizing contribution. Symbols indicate impaction depth: circles = Position C (deep), squares = Position B (medium), triangles = Position A (shallow). In panel (a), all three depth groups fall along the same trend line, indicating that depth does not independently influence the stabilizing contribution beyond its effect on cross-sectional area

**Fig. 15:**
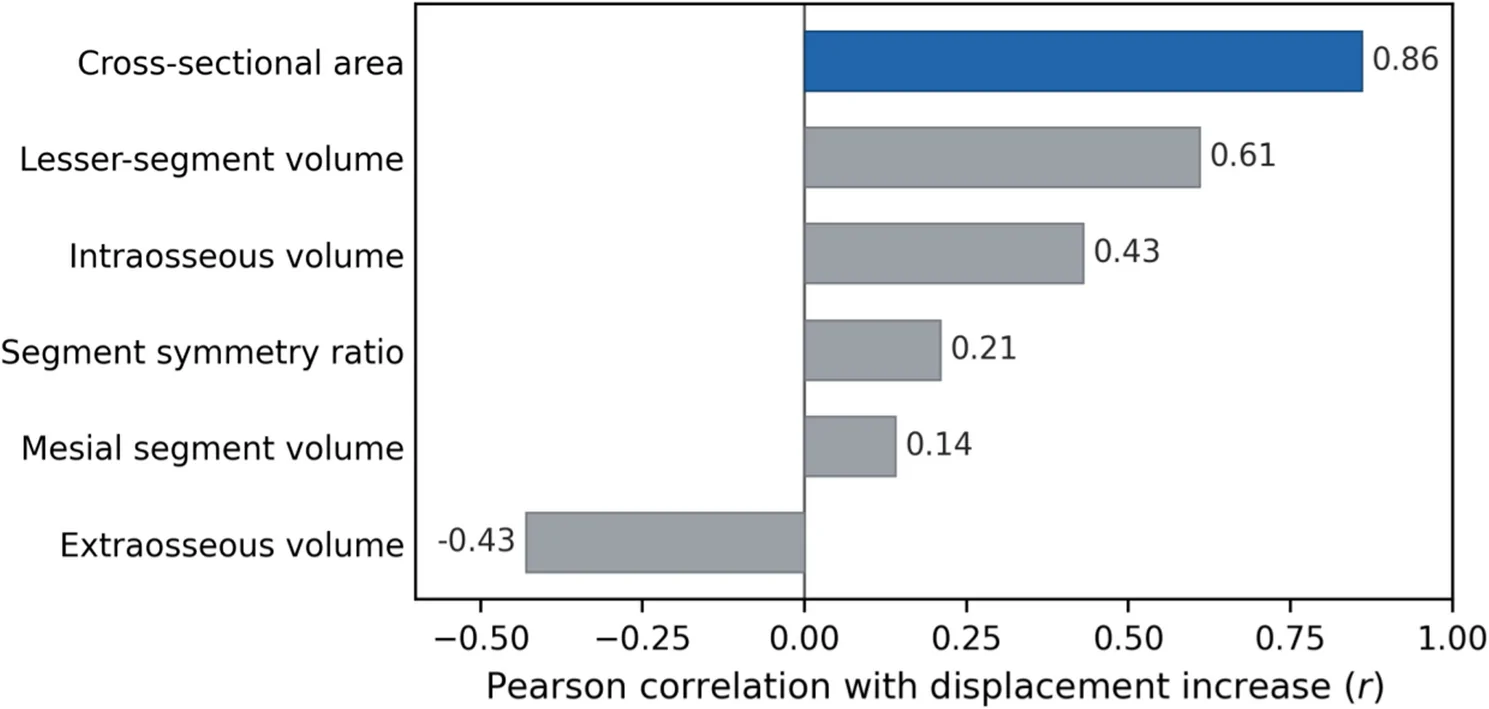
Pearson correlation of geometric variables with the stabilizing contribution of the retained MTM. Each bar shows the Pearson correlation coefficient (*r*) between a candidate geometric variable and the displacement increase upon extraction — the stabilizing contribution of the retained MTM — across the ten configurations. Trans-fracture cross-sectional area (highlighted) showed by far the strongest correlation (*r* = 0.86); no other variable exceeded |*r*| = 0.61. The mesial and distal segments refer to the portions of the tooth on either side of the fracture plane: lesser-segment volume is the smaller of the two intraosseous segment volumes, the segment symmetry ratio their lesser-to-greater ratio, and mesial segment volume the volume of the mesial portion. Intraosseous and extraosseous volume denote total tooth volume inside and outside bone; the negative value for extraosseous volume reflects the mechanically inert, bone-external crown. Cross-sectional area remained the dominant correlate under Spearman’s rank correlation (ρ = 0.79)

To identify which feature of the retained MTM governed its stabilizing contribution, the displacement increase upon extraction was related to a set of candidate geometric variables across the ten configurations (Figs. 14 and 15). The trans-fracture cross-sectional area—the area of the tooth’s cross-section lying on the fracture plane—showed a strong positive association with the stabilizing contribution (*r* = 0.86, ρ = 0.79; Fig. 14a): configurations in which more tooth substance crossed the fracture plane derived a greater benefit from retention. By contrast, the overall volume of the tooth on the mesial side of the fracture showed no meaningful association (*r* = 0.14, ρ = 0.20; Fig. 14b), indicating that the amount of tooth substance per se did not predict stability.

This contrast held across the full panel of geometric variables (Fig. 15). The trans-fracture cross-sectional area was by far the strongest correlate of the stabilizing contribution; no other variable—including lesser-segment volume, the segment symmetry ratio, mesial segment volume, or intraosseous volume—reached an absolute correlation above 0.61, and cross-sectional area remained dominant under Spearman’s rank correlation (ρ = 0.79). Impaction depth did not act as an independent determinant: in Fig. 14a the deep, medium, and shallow configurations all fell along a common trend line, indicating that depth influenced the stabilizing contribution only through its effect on the cross-sectional area presented at the fracture plane. Consistent with this, C-M (a deep, mesioangular impaction) and A-V (a shallow, vertical impaction)—drawn from opposite ends of the depth spectrum—produced two of the smallest stabilizing contributions, in keeping with their similarly small trans-fracture cross-sectional areas, while C-H (horizontal impaction) was the single notable configuration lying above the trend.

## Discussion

The aim of this study was to test two biomechanical hypotheses and to analyze the underlying mechanism through controlled between-group comparisons; accordingly, identical boundary conditions were applied across all 20 models. It should be acknowledged that the model represents a simplification of the clinical situation and does not fully reproduce the in vivo condition. However, precisely because the boundary conditions were identical across all models, this set-up allowed the effect of the MTM—the single variable of interest—on fixation stability to be examined while all other variables were held constant, thereby satisfying the requirement of a mechanistic analysis.

Constraining the condyles with a fixed support is a common approach in mandibular FEA. According to Saint-Venant’s principle [16], the specific manner in which a constraint is applied mainly affects the stress distribution in its immediate vicinity (the condyle and ramus), and its influence on regions remote from the constraint is limited; because the region of interest (ROI) in this study lay at the mandibular angle fracture, at some distance from the constrained region, the specific definition of the boundary conditions had only a limited effect on the results in this region. More importantly, the present study is a qualitative, trend-level analysis, and because identical boundary conditions were applied to all models, the relative trends among configurations would remain largely unchanged even if the boundary conditions were altered as a whole; the trend-level conclusions of this study are therefore unaffected.

During the mesh convergence analysis, we observed that, owing to stress singularities at locations such as sharp geometric corners and contact interfaces, the peak maximum von Mises stress and maximum principal strain continued to increase with mesh refinement and did not converge to a finite value. The failure of peak values to converge at a stress singularity is an inherent numerical feature of linear-elastic finite element models: at an idealized sharp re-entrant corner the theoretical stress tends to infinity [17], so the peak value rises as the mesh is refined, yet this does not indicate that the model is invalid [18]. The conventional approach is to disregard the peak value at the singularity and to evaluate the stress in the surrounding region instead. Because the present study is a qualitative, trend-level analysis that does not interpret absolute peak values, the stress and strain within the ROI were evaluated as element-averaged values with the singular peak nodes excluded; these averaged values converged satisfactorily with mesh refinement and are adequate for the trend analysis.

In reality the mandible is an anisotropic material, whereas the present study assigned isotropic, homogeneous material properties—a simplification commonly adopted in mandibular FEA. Constructing a fully anisotropic model would require a complete set of direction-dependent elastic constants; however, a systematic review [19] has shown that even the most basic mandibular material parameters (elastic modulus and Poisson’s ratio) lack consensus values in the existing literature and vary widely between studies, so that no reliable, standardized anisotropic parameter set is available. Imposing anisotropy would therefore introduce multiple poorly constrained parameters and add uncontrolled variability, contrary to the controlled-variable design of this study. Because identical material properties were assigned across all 20 models and the analysis is based on relative comparison, this simplification cancels systematically in the between-group comparison and does not affect the trend-level conclusions, influencing only the absolute magnitudes; moreover, the cortical bone elastic modulus used in this study falls within the range reported in the aforementioned literature and is therefore a representative choice.

For the measurement of interfragmentary displacement, the directional deformation of each of the two fracture surfaces (i.e., the displacement components along the X, Y, and Z axes) was extracted; the corresponding components of the two surfaces were first subtracted component-wise to obtain the relative-displacement vector, and its magnitude was taken as the interfragmentary displacement.

where the subscripts prox and dist denote the proximal and distal fracture segments, respectively. It should be emphasized that we did not simply subtract the total deformations of the two fracture surfaces. The total deformation is the magnitude (a scalar) of each surface’s displacement vector and reflects only how far that surface has moved from its original position, carrying no directional information; the difference of the two magnitudes (|U_prox| − |U_dist|) is not equal to the magnitude of the relative-displacement vector (|U_prox − U_dist|). The former discards the difference in the directions of motion of the two surfaces and may substantially underestimate—or even entirely mask—the true displacement between the fragments. For example, if one surface moves 0.20 mm along the X axis and the other moves 0.20 mm along the Y axis, their total deformations are equal and their difference is zero, which would be misread as “no relative displacement between the fragments”; in fact the relative-displacement vector of the two surfaces is (0.20, − 0.20, 0), with a magnitude of approximately 0.28 mm, so the fragments have undergone appreciable relative motion. In addition, under loading the entire mandible undergoes a common rigid-body translation; the vector subtraction removes this common component and isolates the true relative motion between the fragments, whereas subtracting the magnitudes does not. Therefore, only by first performing the vector subtraction and then taking the magnitude can the true relative displacement of the fracture fragments be correctly captured (a geometric illustration of this point is provided in the Supplementary Material [HTML file]).

A large body of research has addressed the MTM in the line of mandibular angle fractures, but it has focused mainly on the surgical decision-making and postoperative management of the MTM [20], approaching the problem predominantly from the standpoint of postoperative complications. Recent studies include the management of an injured MTM [21] and the influence of MTM extraction on the choice of fixation pattern [22]. Many investigators have examined whether to extract or retain an MTM in the line of a mandibular angle fracture, most often using postoperative infection as the outcome. A meta-analysis by Bobrowski et al. [23] found no statistically significant difference in postoperative infection between retention and extraction, a conclusion supported by other studies [24]. Other reports, however, indicate that extraction of an asymptomatic MTM increases postoperative complications [25, 26], and more recent work suggests that retention is associated with a lower likelihood of secondary surgery [27]. Taken together with the comprehensive review by Chrcanovic [28], the prevailing view is that an MTM in the line of fracture should be retained, provided that the indications for retention set out in the the AO Foundation guidelines are met [29].

Among the various advantages cited for retaining the MTM, its contribution to the postoperative stability of the fixation is frequently mentioned; however, such statements are largely unsupported by any mechanical evidence. Kumar et al. [30] suggested that a retained MTM may help to stabilize the line of fracture, but this was only a speculative inference, and the reference on which it was based [31] supports merely the view that retention confers a better prognosis, without providing any biomechanical analysis. Yamamoto et al. [32] likewise proposed that the MTM can stabilize the fracture fragments, yet the reference they cited [33] offers no direct evidence for this claim. The most representative of these accounts is the “splinting effect” proposed by Wolujewicz [12]: on the basis of a retrospective radiographic analysis, he suggested that an MTM in the line of fracture may act like a splint—the tooth, embedded in the bone segments on both sides of the fracture and spanning the line of fracture, braces the two fragments and restrains their relative movement, much as an external splint immobilizes a fracture. This conclusion, however, was likewise derived solely from retrospective radiographic observation and was not accompanied by any mechanical analysis.

In summary, although the stabilizing role of an MTM in the line of fracture has been repeatedly noted since Wolujewicz introduced the splinting effect, the relevant research has focused almost entirely on the complication-related outcomes of retention versus extraction; this stabilizing role has remained at the level of clinical conjecture and has never been verified or quantified at the biomechanical level, nor has its key determinant ever been identified.

The finite element results of the present study showed that, under identical boundary conditions, the interfragmentary displacement differed markedly between the MTM-extracted and MTM-retained conditions. We quantified this difference as the percentage increase in displacement upon extraction, using it as a measure of the stabilizing contribution of the MTM in each impaction configuration. In all ten configurations, retention of the MTM reduced the interfragmentary displacement; in other words, an MTM of any classification exerted a mechanical stabilizing effect on the displacement of the fracture fragments. Consistently, the von Mises stresses in the plate and screws and the maximum principal strain in the proximal (ramus–condyle) cortical ROI all increased upon extraction: once the MTM was removed, the load it had previously shared was transferred to the plate, screws, and bone, raising their stresses and strains and thereby indirectly confirming that, when retained, the MTM did carry part of the load. These findings provide finite element biomechanical support for the splinting effect proposed by Wolujewicz—an MTM retained in the line of fracture does function to stabilize the fracture fragments.

Having established that the MTM exerts a stabilizing effect, we next investigated the geometric feature through which this effect is mediated. Wolujewicz observed that MTMs with mesioangular or horizontal impaction were associated with smaller fragment displacement. At the outset of our analysis, we hypothesized an “anchoring effect,” whereby the volume of the tooth lying within the tooth-bearing fragment—that is, the mesial segment of the tooth—would be the decisive factor; for a mesioangular impaction, for example, the crown is inclined mesially against the direction of the occlusal load and might therefore be expected, on structural grounds, to provide greater stability. The results, however, contradicted this expectation: C-M, the configuration with the largest mesial segment volume (348.5 mm³), produced the smallest stabilizing contribution (Fig. 14b), directly refuting the anchoring hypothesis. We then considered a “weak-link” notion (that the smaller of the two intraosseous tooth-segment volumes—the lesser-segment volume—governs stability) and the ratio between the two segment volumes (the segment symmetry ratio); in addition, prompted by the clinical suggestion that a retained tooth aids stability by maintaining contact between the fracture fragments [30], we also included the trans-fracture cross-sectional area of the tooth as a candidate variable.Among these variables, the cross-sectional area showed by far the strongest, and monotonic, correlation with the stabilizing contribution (*r* = 0.86, ρ = 0.79; Fig. 14a), and no other variable approached it (Fig. 15). The lesser-segment volume was only moderately correlated (*r* = 0.61), the intraosseous volume more weakly (*r* = 0.43), the segment symmetry ratio weakly (*r* = 0.21), and the mesial segment volume essentially uncorrelated (*r* = 0.14; Fig. 14b); the bone-external (extraosseous) volume was negatively correlated (*r* = − 0.43). Neither of our two volume-based hypotheses was therefore supported as the primary determinant: the cross-sectional area, rather than the volume of any tooth segment, tracked the stabilizing contribution. Why this is so—and why the volume-based measures nonetheless showed weak-to-moderate correlations—is explained by the load-transfer mechanism considered next.

Why, then, should the cross-sectional area be decisive? From first principles, the fixed fracture surface is bridged by three parallel load paths—the plate (acting as a tension band), the bone-to-bone friction across the apposed fracture surfaces, and, when retained, the MTM—whose stiffnesses add in parallel: k_total = k_plate + k_bone + k_tooth. Under a given load, the interfragmentary displacement equals the load divided by k_total; retaining the MTM introduces an additional spring in parallel, increasing k_total and reducing displacement, whereas extraction removes it. As a structural element spanning the fracture, the MTM forms a connection whose stiffness is governed by its cross-section at the fracture plane. By a structural-mechanics scaling relation, the axial and shear stiffness of such an elastic connector increases with its load-bearing cross-sectional area (k ∝ A); where the load is carried in bending, the dependence is even steeper (approximately k ∝ A²). Because all models in this study used the same tooth and the same fracture-line geometry, the elastic modulus of the tooth and the trans-fracture length scale over which load is transferred were essentially identical across models, so that differences in connection stiffness were governed chiefly by the cross-sectional area at the fracture plane. Moreover, because the elastic modulus of the tooth is approximately 13.6 times that of trabecular bone, this “spring” is intrinsically stiff, so that even a modest cross-section contributes appreciable stiffness. Critically, only the cross-section at the fracture plane lies on the trans-fracture load path; tooth material remote from the plane—whether a large mesial or distal bulk—does not lie on this path and contributes negligibly to the connection stiffness, much as the strength of a chain is set by its narrowest link rather than by the size of the masses at either end.

This mechanism accounts for the entire pattern in Fig. 15. It explains why the mesial segment volume failed to predict the stabilizing contribution (*r* = 0.14; Fig. 14b) and why the bone-external portion of the crown, lying off the load path, was negatively correlated (extraosseous volume, *r* = − 0.43). It likewise explains why the volume-based measures correlated only partially. A tooth presenting a larger cross-section at the fracture plane generally also has a larger bulk straddling that plane, so measures such as lesser-segment volume (*r* = 0.61) and intraosseous volume (*r* = 0.43) partly track the cross-sectional area. Because these are volumes aggregated along the length of the tooth rather than the load-bearing area at the plane itself, they reflect the cross-section only indirectly and therefore correlate more weakly than the cross-sectional area itself (*r* = 0.86). Seen in this way, our initial “weak-link” intuition was right in spirit but wrong in its object: the true rate-limiting “narrowest link” is the cross-section at the fracture plane, not the smaller of the two volume segments.

Consistent with this mechanism, the conventional impaction parameters appear to act only indirectly. In Fig. 14a, configurations spanning all three impaction depths fall along a single cross-sectional-area trend, with no systematic separation between the depth groups; the two configurations with the smallest stabilizing contributions—C-M (deep) and A-V (shallow)—lie at opposite ends of the depth spectrum yet behave similarly, consistent with their similarly small cross-sectional areas. This indicates that impaction depth influences the stabilizing contribution chiefly through the cross-section it produces at the fracture plane, rather than independently. Taken together, and under the controlled conditions of this study—in which the trans-fracture load-transfer length scale was held constant—these results provide a quantitative mechanical basis for Wolujewicz’s splinting effect and identify the trans-fracture cross-sectional area of the tooth, rather than its angulation or impaction depth per se, as the key determinant of that effect.

### Limitations

This study has several limitations. First, as a theoretical biomechanical analysis based on the finite element method, it is a simplification of the clinical situation and cannot fully reproduce the in vivo environment: the model was constructed from the anatomy of a single healthy adult, the materials were assigned isotropic and homogeneous properties, and the loading and boundary conditions were idealized. These simplifications are widely adopted in comparable mandibular finite element studies; more importantly, because all twenty models shared identical simplifications and settings, the associated errors cancel systematically between groups, so that the trend-level conclusions drawn from relative comparison are not materially affected and only the absolute magnitudes are influenced.

Second, although solution-level verification (mesh convergence analysis) was performed, the study was not validated against physical specimens. The mechanical behavior and relative trends reported here remain to be corroborated by in vitro mechanical testing or cadaveric studies. It should be noted that finite element analysis can characterize the mechanical response of a structure but cannot simulate the complex biological cascade of fracture healing; the present conclusions should therefore be understood as a relative assessment of mechanical stability rather than a direct prediction of clinical outcomes.

Third, and pertinent to the core mechanism of this study, the length scale over which the tooth transfers load across the fracture plane was held constant across all models (the same tooth and the same fracture-line geometry). It was precisely under this controlled condition that differences in connection stiffness could be attributed to the cross-sectional area at the fracture plane alone (k ∝ A), allowing the cross-sectional area to be identified as the key geometric determinant. In clinical reality, this length scale varies with individual anatomy and fracture-line orientation. More generally, the stiffness of an elastic connector depends on its load-bearing cross-sectional area relative to the length over which load is transferred (e.g., k ∝ A/L), in which the cross-sectional area remains a positive determinant whose direction does not change. Whether the cross-sectional area alone remains sufficient to predict the stabilizing contribution when this length scale is allowed to vary cannot, however, be determined from the present study, because that length scale did not vary here and its independent effect was not examined. Across the population of impacted teeth, the cross-sectional area at the fracture plane typically varies far more than the trans-fracture length scale, which suggests that the cross-sectional area is likely to remain dominant. This is a plausibility argument based on anatomical considerations, however, not a conclusion supported by the present data; it awaits verification by parametric, multi-sample studies that treat the depth, angulation, and mesiodistal position of the tooth—and the resulting cross-section and load-transfer length—as continuous parameters.

In addition, this study employed only a single fixation method (a single Champy miniplate along the external oblique ridge) and a single fracture-line configuration; it did not compare alternative fixation strategies such as double plates, reconstruction plates, lag screws, or locking systems, nor did it cover fracture lines of differing orientation, and the relative effects may differ with the fixation method or the fracture-line configuration.

Despite these limitations, the present study provides, under clearly defined controlled conditions, the first quantitative mechanical basis for the stabilizing role of an MTM retained in the line of fracture, and identifies the trans-fracture cross-sectional area of the tooth—rather than the conventional angulation- or depth-based classifications—as the key geometric determinant of this stabilizing contribution. This finding redirects clinical attention from the conventional impaction classifications toward the load-bearing cross-section at the fracture plane and points the way toward future parametric, multi-sample, and experimental validation studies.

## Conclusion

Under the conditions of this finite element model, retention of a mandibular third molar in the line of a mandibular angle fracture reduced the interfragmentary displacement in all impaction configurations studied, indicating a consistent stabilizing effect on the fixation; this provides biomechanical support for the “splinting effect” previously proposed on clinical grounds.

Under a controlled trans-fracture load-transfer length scale, the magnitude of this stabilizing effect was governed chiefly by the cross-sectional area of the tooth at the fracture plane rather than by its impaction angulation or depth in themselves; the conventional angulation- and depth-based classifications appear to act mainly indirectly, through the cross-section they produce at the fracture plane. Generalization of this relationship to settings with a variable length scale warrants further investigation.

These mechanical findings suggest that, when the potential stabilizing value of an MTM in the line of fracture is considered, the load-bearing cross-section of the tooth at the fracture plane may be more mechanically relevant than the conventional impaction classifications and merits attention in future clinical research; however, until prospective clinical evidence is available, these results should not be used directly to guide clinical decision-making.

## Supplementary Information

Supplementary Material 1.

Supplementary Material 2.

## Data Availability

The finite element models generated during this study are available from the corresponding author on reasonable request.

## Abbreviations

MTM: Mandibular third molar
FEA: Finite element analysis
ROI: Region of interest
CBCT: Cone-beam computed tomography
DICOM: Digital Imaging and Communications in Medicine
STL: Standard tessellation language
Impaction classification codes (Pell and Gregory depth × Winter angulation: e.g., C-H denotes a horizontally impacted MTM in Position C)
A, B, C: Pell and Gregory positions
M: Mesioangular (Winter)
D: Distoangular (Winter)
V: Vertical (Winter)
H: Horizontal (Winter)
