# Supplementary material for: The influence of mandibular third molar classification on fixation stability of mandibular angle fractures: a biomechanical finite element analysis
Source: BMC Oral Health. 2026 Jul 20;26:2057. doi: 10.1186/s12903-026-09329-9 (PMC13678975; doi:10.1186/s12903-026-09329-9)
Supplement: Supplementary file 1 [file 12903_2026_9329_MOESM2_ESM.html]

Relative displacement — interactive


# Why subtracting two total displacements fails

Two points start at the same spot, then each moves off by its own displacement — **U₁** and **U₂**. Their relative displacement is the gap between the final tips: **|U₁ − U₂|**. Subtracting the lengths, |U₁| − |U₂|, only compares how far each traveled and ignores direction. Drag the slider to change the angle between them: the length‑subtraction stays frozen while the true relative displacement moves.

Why two total displacements cannot be subtracted
Two displacement arrows start from a common point with fixed lengths; the difference of their lengths stays constant, while the relative-displacement arrow joining the two tips changes with the angle between them.


U₁
U₂
ΔU

Length |U₁|

4.00

Length |U₂|

3.00

Subtract lengths (wrong) · |U₁| − |U₂|

1.00

Relative displacement (correct) · |U₁ − U₂|

3.09

Angle between U₁ and U₂

50°

The two answers agree only when the displacements point the same way (angle 0°). The right way, in components: subtract first, then take the magnitude — `|U₁ − U₂| = √[(ΔUx)² + (ΔUy)² + (ΔUz)²]`. Taking each magnitude first and then subtracting throws the direction away.
